# Synthesis and Characteristics of FePt Nanoparticle Films Under In Situ-Applied Magnetic Field

**DOI:** 10.1186/s11671-016-1543-1

**Published:** 2016-07-11

**Authors:** Xu Qian, Mo-Yun Gao, Ai-Dong Li, Xiao-Yu Zhou, Xiao-Jie Liu, Yan-Qiang Cao, Chen Li, Di Wu

**Affiliations:** National Laboratory of Solid State Microstructures and Department of Materials Science and Engineering, College of Engineering and Applied Sciences, Collaborative Innovation Center of Advanced Microstructures, Nanjing University, Nanjing, 210093 People’s Republic of China

**Keywords:** L1_0_-phase FePt, Chemical solution synthesis, Applied magnetic field, C-axis oriented, Magnetic anisotropy

## Abstract

In situ external magnetic field was applied during the synthesis of FePt nanoparticles via a chemical solution method. FePt nanoparticle films were prepared on Si by a drop-coating method with and without a magnetic field. Annealing at 700 °C in reductive atmosphere was explored to obtain ferromagnetic FePt L1_0_ phase. The effect of in situ-applied magnetic field on the structure, morphology, and magnetic properties of FePt nanoparticle films was characterized. It is found that the applied magnetic field during the chemical synthesis of FePt nanoparticles plays a key role in the crystallinity and magnetic property of FePt nanoparticle films. As-synthesized FePt nanoparticles under the magnetic field are monodispersed and can be self-assembled over a larger area by a dropping method. The applied magnetic field during the synthesis of FePt nanoparticles not only significantly improves the nanoparticles’ c-axis preferred orientation but also benefits the phase transition of FePt nanoparticles from face-centered cubic to face-centered tetragonal structure during the annealing process. The FePt nanoparticle films derived under magnetic field also show some magnetic anisotropy.

## Background

With the rapid development of magnetic recording technique, the superparamagnetic effect becomes the bottleneck to further increase magnetic storage density. The ferromagnetic L1_0_ FePt assemblies with face-centered tetragonal (fct) structure has extremely high magnetocrystalline anisotropy, good chemical stability, and resistance to oxidation, regarded as the most promising candidate for ultra-high-density magnetic recording media [1–3]. Meanwhile, FePt nanoparticles and their multifunctional surfaces have also shown great potentials in biomedical applications such as multimodality imaging probes and target-specific drug/gene delivery [1, 4].

Chemical solution method has become an attractive route to obtain FePt nanoparticles with the controllable size, well-defined shape, and ordered monolayer assemblies since Sun et al. made great success in preparing monodisperse FePt nanoparticles [5]. Based on this work, a lot of studies have been conducted to explore and optimize the synthesis of FePt nanoparticles, such as modifying fabrication methods [6–10], optimizing assembly methods [7, 11–14], and fabricating FePt one-dimensional nanorods/nanowires [15–18].

For example, elements such as Ag [19], Au [20], and Sb [21] with low surface energy were doped into FePt nanoparticles to decrease the phase transition temperature of FePt from face-centered cubic (fcc) to fct structures. However, the morphology of FePt nanoparticles became uncontrolled, and self-assembled arrays over a large area were destroyed after doping. A series of inorganic core-shell structures such as ZnO [22, 23], MnO [24], NiO [25], and SiO_2_ [26] covering on FePt nanoparticles have been synthesized to obtain multifunctional magnetic nanoparticles. In addition, polymer templating [8, 27], micellar approach with SiO_2_ thin film capping [28], direct synthesis [29, 30], and salt-matrix technique [31] have also been attempted. Recently, our group explored the combination of self-assembled FePt nanoparticles and atomic layer-deposited Al_2_O_3_ capping for fabrication of patterned magnetic nanocomposites with improved the coercivity and stability of FePt nanoparticles under high-temperature annealing [32]. In addition, sol-gel-derived oxide matrixes also prevented FePt nanoparticles from sintering and aggregation [33, 34].

In this work, we reported that in situ magnetic field was applied during the chemical solution synthesis process of FePt nanoparticles. And the drop-coating process was explored to form FePt nanoparticle films with and without a magnetic field. Under a magnetic field, as-synthesized FePt nanoparticles are monodispersed and can be self-assembled over a larger area by a dropping method. As-prepared FePt nanoparticle films were then annealed at 700 °C for 60 min in forming gas (7 % H_2_ + 93 % Ar) to obtain the L1_0_ phase of FePt. It is revealed that an applied magnetic field during chemical synthesis not only significantly improves the c-axis preferred orientation but also benefits the phase transition of FePt nanoparticles from fcc to fct structures. The FePt nanoparticle films chemical-synthesized under the magnetic field also show some magnetic anisotropy.

## Methods​

### Synthesis of FePt Nanoparticles

**Fig. 1:**
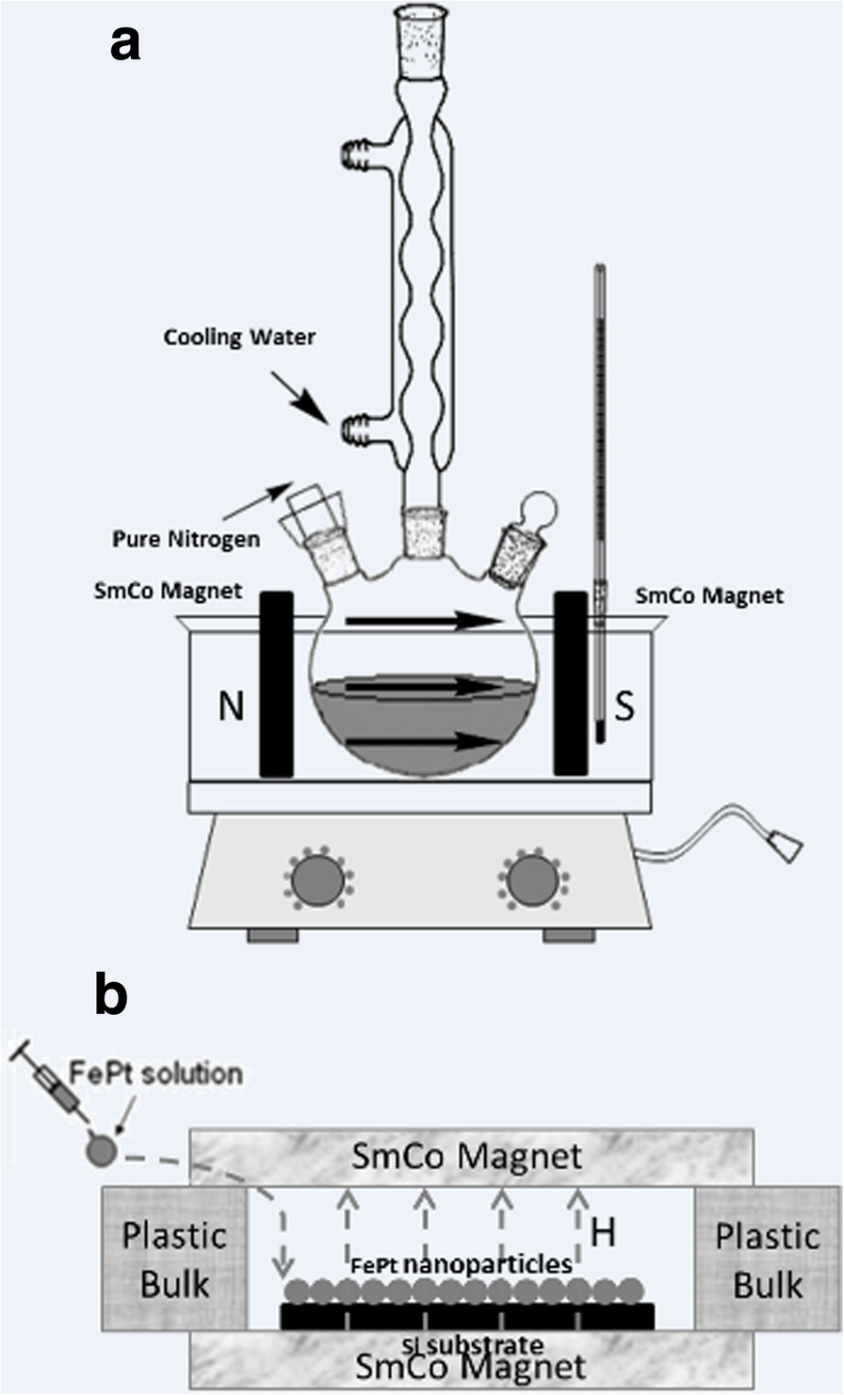
The apparatus schematics of **a** chemical synthesis of FePt nanoparticles and **b** drop-coating process for FePt nanoparticle films under in situ-applied magnetic field

FePt nanoparticles were synthesized through a modified polyol process under a gentle flow of pure nitrogen (N_2_) [32]. Typically, the FePt nanoparticles were prepared via chemical reduction of Pt(acac)_2_ and thermal decomposition of Fe(CO)_5_ in the presence of oleic acid (OA) and oleylamine (OAm) at 220 °C under in situ-applied magnetic field, as shown in Fig. 1a. Two SmCo permanent magnets (5 × 5 cm^2^) were placed vertically to yield external magnetic field H of 6000 Oe.

In a typical procedure, 49.4 mg of Pt(acac)_2_ was mixed with 20 mL of phenyl ether under nitrogen flow. The mixture was heated to 50 °C and stirred until the platinum source dissolved in the solvent completely. Then the mixed solution was heated to 150 °C, and 80 μL of Fe(CO)_5_, 40 μL of OA, and 42.5 μL of OAm were added step by step under in situ-applied magnetic field with continuous stream of nitrogen. Finally, the solution was heated up to 220 °C at the rate of 5 °C/min and refluxed for 30 min under the nitrogen protection. After the black solution cooling down to room temperature naturally, 50 μL of OA, 50 μL of OAm, and absolute ethanol were added into the mixture to a total volume of 80 mL in a clean beaker. The black products were then precipitated by centrifugation at 8000 rpm for 10 min, and the supernatant solution was discarded. The precipitate was then dissolved in 10 mL of hexane and precipitated again in 40 mL of absolute ethanol by centrifugation. The black FePt nanoparticles were obtained by repeating the separation process for 2~3 times. The FePt nanoparticles were dispersed in 6 mL of octane and stored in a brown glass bottle under the nitrogen conditions. For comparison study, we also prepared the control samples of FePt nanoparticles using the same processing without an applied magnetic field.

### Preparation of FePt Nanoparticle Films

Assembled FePt nanoparticles on the HF-treated n-Si (100) substrates (1.0 × 1.0 cm^2^) were prepared by dropping a drop of FePt solution (FePt nanoparticles dispersed in octane with concentration of 2 mg/mL). The FePt nanoparticle films were first dried at room temperature and then heated to 120 °C for 2 h in a baking oven to remove organic solvent completely. In situ external magnetic field was applied during the drop-coating process to form FePt nanoparticle films on Si, as illustrated in Fig. 1b. Two SmCo permanent magnets were placed horizontally to produce a magnetic field by inserting 1-cm-high plastic spacer.

Three kinds of samples with different external magnetic field conditions during the chemical synthesis process for FePt nanoparticles and the drop-coating process for FePt nanoparticle films were listed in Table 1. The prepared FePt nanoparticle films were then annealed at 700 °C for 60 min in forming gas (7 % H_2_ + 93 % Ar) with a ramp rate of 5 °C/min to obtain an ordered ferromagnetic fct-FePt phase.

**Table 1:** The applied magnetic field conditions of different samples during the chemical synthesis process for FePt nanoparticles and the drop-coating process for FePt nanoparticle films

Sample	Applied magnetic field	
	Chemical synthesis	Drop-coating
1^#^	No	No
2^#^	Yes	No
3^#^	Yes	Yes

### Characterization

The structure and crystalline phase were characterized by means of X-ray diffraction (XRD, D/max 2000, Rigaku) using Cu Kα radiation (*λ* = 1.5406 Å) operated at 40 kV and 40 mA, respectively. The morphology and microstructure of various samples were characterized using a transmission electron microscopy (TEM, Tecnai G^2^ F20 S-twin, FEI) operating at 200 kV. The compositions of all the samples were analyzed by an energy dispersive X-ray spectroscope (EDS) attached to a field emission scanning electron microscope (FESEM, Zeiss). Magnetic properties of the fct-FePt were measured by a superconducting quantum interference device (SQUID, MPMS XL-7, Quantum Design) with a maximum field of 35 kOe.

## Results and Discussion

**Fig. 2:**
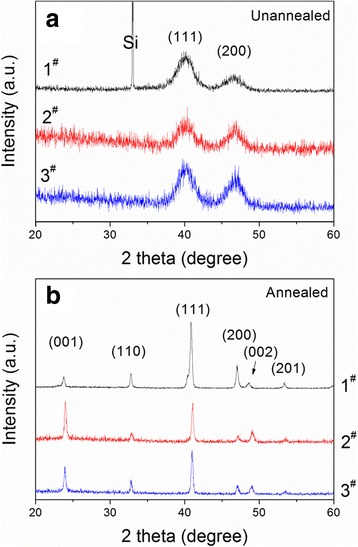
The XRD patterns of **a** unannealed and **b** 700 °C annealed FePt nanoparticle films with different magnetic field conditions

The effect of applied external magnetic field on the crystalline phase and orientation of FePt nanoparticle films on Si have been examined by XRD. Figure 2a, b shows the XRD patterns of unannealed and annealed FePt nanoparticle films on Si under different magnetic conditions corresponding to 1^#^, 2^#^, and 3^#^ samples. In Fig. 2a, all unannealed samples have two broad peaks at 40.3° and 46.9°, assigned to the (111) and (200) lattice planes, respectively, from superparamagnetic fcc-FePt nanoparticles. All the samples exhibit similar average grain size of ~4.1 ± 0.3 nm calculated by the Scherrer equation, indicating that the in situ-applied magnetic field has no effect on the grain size of the FePt nanoparticles from chemical synthesis and drop-coating process. Another noticeable feature is that the relative intensity ratio of I_(200)_/I_(111)_ peaks of 0.89 and 0.76 in the 2^#^ and 3^#^ samples, respectively, with an applied magnetic field is stronger than that of 0.46 in the 1^#^ sample without an applied magnetic field, as seen in Table 2. This indicates that FePt nanoparticles derived from chemical synthesis under in situ-applied magnetic field tend to align perpendicular to the (100) crystal plane. Whereas the applied magnetic field during drop-coating process for the FePt nanoparticle films has no obvious influence on the orientation of the 2^#^ and 3^#^ samples.

**Table 2:** The unannealed I_(200)_/I_(111)_, annealed I_(001)_/I_(111)_, D_(001)_, and S of c-axis orientation degree and chemical ordering of 1^#^, 2^#^, and 3^#^ samples under different magnetic field conditions before and after annealing

Sample	Unannealed I_(200)_/I_(111)_	Annealed I_(001)_/I_(111)_	Annealed D_(001)_	Annealed S
1^#^	0.46 ± 0.03	0.19 ± 0.02	0.63 ± 0.07	0.72 ± 0.02
2^#^	0.89 ± 0.03	1.04 ± 0.03	3.47 ± 0.10	0.76 ± 0.03
3^#^	0.76 ± 0.11	0.65 ± 0.02	2.17 ± 0.07	0.77 ± 0.02

After 700 °C annealing in forming gas, the Bragg peaks of (001), (110), (002), and (201) appear in the XRD patterns of Fig. 2b, suggesting the phase transition from disordered fcc to ordered ferromagnetic fct. It is easily observed that the peak intensity of (001) and (002) from the 2^#^ and 3^#^ samples, respectively, is much stronger than that from the 1^#^ sample. Especially for the 2^#^ sample, the (001) peak intensity has exceeded the usual strongest (111) peak one (PDF #01-43-1359), and the intensity of the (002) peak split from the (200) peak is also higher than the (200) peak one. It means that after annealing, the fct-FePt nanoparticle films from the applied magnetic field synthesis process exhibit a c-axis preferred orientation, i.e., fct-FePt nanoparticles aligning along the c-axis—the easy axis of magnetization perpendicular to the surface of films [35]. In situ-applied magnetic field during the chemical synthesis process has evidently improved the c-axis preferred orientation of annealed FePt nanoparticle films.

The c-axis preferred orientation degree (D_(001)_) of fct-FePt is defined as follows [36]:

1 $$ {\mathrm{D}}_{(001)} = {\left({\mathrm{I}}_{(001)}/{\mathrm{I}}_{(111)}\right)}_{\mathrm{measured}}/{\left({\mathrm{I}}_{(001)}/{\mathrm{I}}_{(111)}\right)}_{\mathrm{standard}} $$

where the I_(001)_/I_(111)_ standard value of 0.3 is obtained from diffraction patterns of fct-FePt powders with random orientation, while the I_(001)_/I_(111)_ measured values can be calculated from the XRD patterns of the 1^#^, 2^#^, and 3^#^ annealed samples.

The chemical ordering parameter S is introduced to determine the degree of chemical ordering of FePt nanoparticle films quantitatively, defined as follows [37, 38]:

2 $$ {\mathrm{S}}^2 = \left[1\hbox{-} {\left(\mathrm{c}/\mathrm{a}\right)}_{\mathrm{measured}}\right]/\left[1\hbox{-} {\left(\mathrm{c}/\mathrm{a}\right)}_{\mathrm{standard}}\right] $$

where c and a are the lattice constants for the fct-FePt, evaluated from the (001) and (110) peaks of the XRD patterns, respectively. The c/a measured values can be calculated for the partially ordered phase. For the fully ordered-phase FePt, the c/a standard value is 0.9639 based on the XRD card of PDF #01-43-1359.

The related data of c-axis orientation degree and chemical ordering of the 1^#^, 2^#^, and 3^#^ samples under different magnetic conditions before and after annealing are summarized in Table 2, including unannealed I_(200)_/I_(111)_, annealed I_(001)_/I_(111)_, D_(001)_, and S.

It is easily seen from Table 2 that the 2^#^ and 3^#^ samples with applied magnetic field during chemical synthesis show some a-axis preferred orientation than the 1^#^ sample without applied magnetic field before anneal. After annealing, the 2^#^ and 3^#^ samples with an applied magnetic field during chemical synthesis exhibit a significant [001] preferred orientation with higher D_(001)_ values of 3.47 and 2.17, respectively, whereas the D_(001)_ value of the 1^#^ sample without an applied magnetic field is only 0.19. In addition, an applied magnetic field during the synthesis of FePt nanoparticles also enhances the chemical ordering parameter S of the 2^#^ and 3^#^ samples and benefits the phase transition of FePt nanoparticles from fcc to fct structure during the annealing process.

Why does the applied magnetic field during the chemical synthesis of FePt nanoparticles finally produce c-axis preferred orientation fct-FePt nanoparticle films on Si? It might be related to the nucleation anisotropy induced by an external magnetic field during the chemical synthesis of FePt nanoparticles. The applied magnetic field changes the nucleation barrier of a different orientation, leading to an enhanced a-axis orientation in superparamagnetic FePt particles. During high-temperature annealing, the a-axis orientation fcc nucleation easily induces and yields c-axis orientation fct grains.

**Fig. 3:**
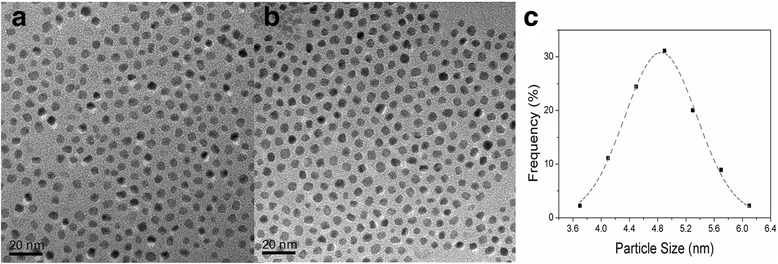
The grain morphology and size distribution of FePt nanoparticles. The TEM images of FePt nanoparticles synthesized with (**a**) and without (**b**) in situ-applied magnetic field. **c** The particle size distribution curve for as-prepared FePt nanoparticles under magnetic field from Fig. 3b

As-synthesized FePt nanoparticles with and without an applied in situ magnetic field are both spherical shapes, as shown in Fig. 3a, b. The particles have an average size of 4.8 nm with better monodispersibility, measured from the TEM images, which is similar to the calculated value of ~4.1 nm from the XRD patterns using the Scherrer equation. The measured particle sizes from more than 100 FePt particles from the TEM image of Fig. 3b are fitted into the Gaussian curves (dashed line, Fig. 3c), and the calculated standard deviation is 0.6 nm. And a typical hexagonal close-packed structure can be recognized in Fig. 3a, b, indicating the FePt nanoparticles can keep their monodispersity with a better self-assembly pattern even if synthesized under in situ magnetic field. An analysis by EDS attached to SEM records the measured average Fe:Pt ratios of 50.9:49.1 and 52.8:47.2 for FePt nanoparticles with and without an applied magnetic field, respectively. Both are close to 1:1, suggesting that in situ-applied magnetic field during chemical synthesis has no obvious impact on the chemical composition of FePt nanoparticles. Some literature has reported that when the Fe:Pt ratio is represented by Fe_*x*_Pt_100 − *x*_ (40 ≤ x ≤ 60), nanoparticles can be transformed to ferromagnetic fct structure without the secondary phase such as Pt_3_Fe or Fe_3_Pt [39, 40].

**Fig. 4:**
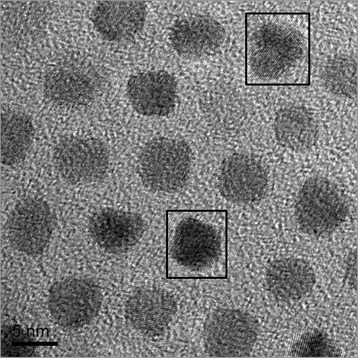
The HRTEM image of as-synthesized FePt nanoparticles under applied magnetic field

A typical high-resolution HRTEM image of as-synthesized FePt nanoparticles synthesized under in situ magnetic field is shown in Fig. 4. The (200) and (111) lattice planes can be well recognized with measured average lattice spacing of 1.98 and 2.25 Å, corresponding to the standard lattice spacing of 1.91 and 2.20 Å, respectively. All FePt nanoparticles are single crystals. Among them, two nanoparticles denoted by □ show two mutually perpendicular planes, implying that these two nanoparticles align along c-axis orientation whose direction is perpendicular of carbon film.

**Fig. 5:**
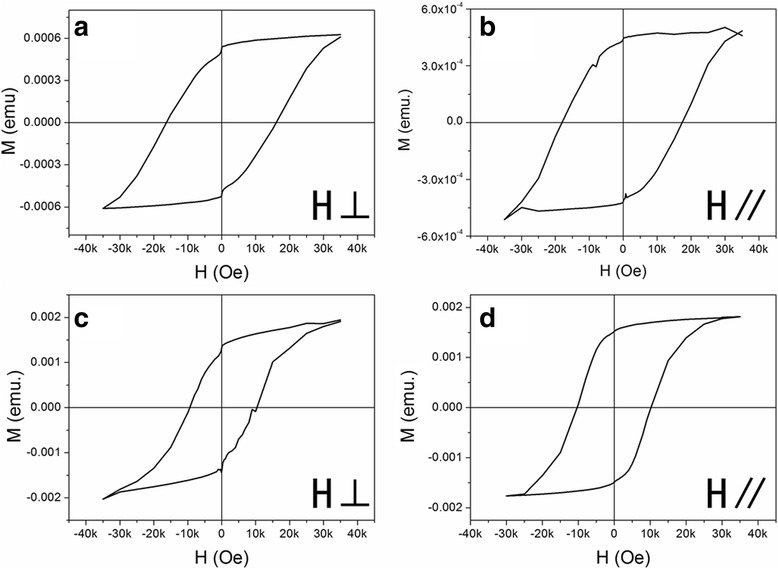
The hysteresis loops of FePt nanoparticle films for the 2^#^ (**a**, **b**) and 3^#^ (**c**, **d**) samples. The applied magnetic field during measuring is perpendicular to the film with denotation of *H⊥* and parallel to the film with *H∥*

The FePt nanoparticle films synthesized under magnetic field also show some magnetic anisotropy which was characterized by SQUID after 700 °C annealing. The in-plane coercivity and out-plane coercivity are represented with Hc,_∥_ and Hc,_⊥_, respectively. The ratio value (*h*) of Hc,_∥_/Hc,_⊥_ can be used to determine the orientation degree of magnetic moments (M). If h = 1, it means that the fct-FePt nanoparticle films are completely random oriented; h > 1, the M is in-plane oriented; h < 1, the M is out-of-plane oriented. Figure 5 plots the hysteresis loops of the 2^#^ and 3^#^ samples measured under an applied magnetic field parallel to or perpendicular to the films. The annealed 2^#^ sample with an applied magnetic field during chemical synthesis and without a magnetic field during drop-coating has the Hc,_∥_ and Hc,_⊥_ of 17.8 and 16.2 kOe with a remanence ratio of 0.89 and 0.82, respectively. The *h* value of 1.10 indicates some degree of in-plane oriented. Correspondingly, the annealed 3^#^ sample with an applied magnetic field during chemical synthesis and drop-coating has the Hc,_∥_ and Hc,_⊥_ of 10.2 and 9.8 kOe with remanence ratio of 0.84 and 0.682, respectively. The calculated *h* value of 1.04 also shows slight in-plane oriented. That is to say, the in-plane coercivity and corresponding remanence ratio are larger than the out-of-plane ones.

## Conclusions

In summary, in situ magnetic field was applied during the synthesis of FePt nanoparticles via a chemical solution method. FePt nanoparticle films were formed on Si by a drop-coating method with and without a magnetic field. The influence of in situ-applied magnetic field on the structure, morphology, and magnetic properties of FePt nanoparticle films was characterized deeply. Although in situ magnetic field has no obvious impact on the FePt nanoparticles’ morphology and chemical composition, it is revealed that an applied magnetic field during the synthesis of FePt nanoparticles not only significantly improves the nanoparticles’ c-axis preferred orientation with a larger D_(001)_ of 3.47 but also benefits the phase transition of FePt nanoparticles from fcc to fct structure. The FePt nanoparticle thin films synthesized under a magnetic field also show some magnetic anisotropy. In addition, as-synthesized FePt nanoparticles under a magnetic field are monodispersed and can be self-assembled over a larger area by a dropping method.
